# Interictal Spike and Loss of Hippocampal Theta Rhythm Recorded by Deep Brain Electrodes during Epileptogenesis

**DOI:** 10.3390/s22031114

**Published:** 2022-02-01

**Authors:** Xiaoxuan Fu, Youhua Wang, Abdelkader Nasreddine Belkacem, Yingxin Cao, Hao Cheng, Xiaohu Zhao, Shenghua Chen, Chao Chen

**Affiliations:** 1 State Key Laboratory of Reliability and Intelligence of Electrical Equipment, Hebei University of Technology, Tianjin 300130, China; 201711401003@stu.hebut.edu.cn (X.F.); wangyouhua@hebut.edu.cn (Y.W.); 202031404083@stu.hebut.edu.cn (Y.C.); 202031403008@stu.hebut.edu.cn (H.C.); 202031403034@stu.hebut.edu.cn (X.Z.); 2 Key Laboratory of Electromagnetic Field and Electrical Apparatus Reliability of Hebei Province, Hebei University of Technology, Tianjin 300130, China; 3 Athinoula A. Martinos Center for Biomedical Imaging, Department of Radiology, Massachusetts General Hospital/Harvard Medical School, Boston, MA 02114, USA; 4 Department of Neuroscience, Medical University of South Carolina, Charleston, SC 29464, USA; 5 Department of Computer and Network Engineering, College of Information Technology, United Arab Emirates University, Al Ain P.O. Box 15551, United Arab Emirates; belkacem@uaeu.ac.ae; 6 Key Laboratory of Complex System Control Theory and Application, Tianjin University of Technology, Tianjin 300384, China

**Keywords:** interictal spike, theta rhythm, epileptogenesis, temporal lobe epilepsy, intracortical microelectrodes

## Abstract

Epileptogenesis is the gradual dynamic process that progressively led to epilepsy, going through the latent stage to the chronic stage. During epileptogenesis, how the abnormal discharges make theta rhythm loss in the deep brain remains not clear. In this paper, a loss of theta rhythm was estimated based on time–frequency power using the longitudinal electroencephalography (EEG), recorded by deep brain electrodes (e.g., the intracortical microelectrodes such as stereo-EEG electrodes) with monitored epileptic spikes in a rat from the first region in the hippocampal circuit. Deep-brain EEG was collected from the period between adjacent sporadic interictal spikes (lasting 3.56 s—35.38 s) to the recovery period without spikes by videos while the rats were performing exploration. We found that loss of theta rhythm became more serious during the period between adjacent interictal spikes than during the recovery period without spike, and during epileptogenesis, more loss was observed at the acute stage than the chronic stage. We concluded that the emergence of the interictal spike was the direct cause of loss of theta rhythm, and the inhibitory effect of the interictal spike on ongoing theta rhythm was persistent as well as time dependent during epileptogenesis. With the help of the intracortical microelectrodes, this study provides a temporary proof of interictal spikes to produce ongoing theta rhythm loss, suggesting that the interictal spikes could correlate with the epileptogenesis process, display a time-dependent feature, and might be a potential biomarker to evaluate the deficits in theta-related memory in the brain.

## 1. Introduction

Complain of cognitive deficits are usual in either newly diagnosed patients or patients with long-standing seizures. Interictal activities (IAs), pathological discharges occurring between seizures, and important diagnostic biomarkers have been correlated with some types of cognitive deficits or memory deficits [1,2,3]. Epileptogenesis is defined as the process during which changes occur in the brain after a precipitating injury or insult that results in epilepsy. It is crucial to study the temporal character caused by abnormal discharges during two successive stages, including latent/early and chronic/later stages. Most patients have epilepsy and are at the chronic stage during epileptogenesis. Only the animal model could experience the latent stage. In a patient, intracortical electroencephalography, such as stereo-electroencephalograms (SEEGs), could only record IAs, while in an animal model, the interictal-like activities (ILAs) can be recorded by deep-brain EEG (i.e., SEEGs) in the latent/early stage during epileptogenesis. Research showed that either an ILA or an IA are related to the neural circuits, and appeared in different structures, independently or in a synchronous way. Recently, IAs of generative network and propagation pathways are determined to predict the post-surgery outcome [4]. In most common cases, the epileptogenic zone appeared in both ipsi- and contralateral brain hemispheres, between interictal periods, and before seizures [5,6]. Its morphological feature was a spike immediately followed by a wave, which explained why an ILA or an IA was called “spike and wave”. This wave correlated with the extent of impairment, rather than seizure frequency, severity, and etiology [7]. However, many lines of evidence of the negative effects of both etiology and seizures reported an important controversy was whether ILAs or IAs were harmless biomarkers to evaluate the cognitive deficits [8,9]. 

Different from the non-invasive electrodes of scalp EEG, the electrodes of intracranial EEG (iEEG) need to be implanted into the deep brain, including the cortical surface electroencephalogram (ECoG) and the intracortical electroencephalography, such as SEEGs. They are mainly made of silver, stainless steel, or platinum–iridium alloy. The inner diameters of electrodes are varied from 0.5 mm to 3 mm and strip shaped (commonly used as 5~8 leads) or grid shaped (disc-shaped electrodes with a diameter of 5~10 mm, buried on silicon film, usually 10 mm apart between the electrodes). SEEG electrode implantation plan is based on MRI, and its signal is a direct recording of cortical signals in the brain area, so the SEEG signal itself has high temporal resolution and spatial resolution, as well as a high ratio to noise [10].

Cognitive deficits and IAs or ILAs share the same neuronal networks, involved in physiological activities, cognitive and behavioral functions, as well as IA or ILA genesis. To produce physiological activities, the network generates oscillations, such as theta rhythm (4–8 Hz), which is prominent during voluntary movements, attention, rapid eye movement (REM) sleep, arousals from sleep, and calm wakefulness, as well as transcranial alternating current stimulation (tACS) behavior [11]. Theta oscillations might themselves influence, coordinate collections of neurons, or assemble neurons in hippocampal networks. Such neuronal networks would be rewritten by IAs or ILAs; consequently, theta rhythm could be alerted in TLE, the most common form of partial epilepsy [12,13,14].

**Figure 1:**
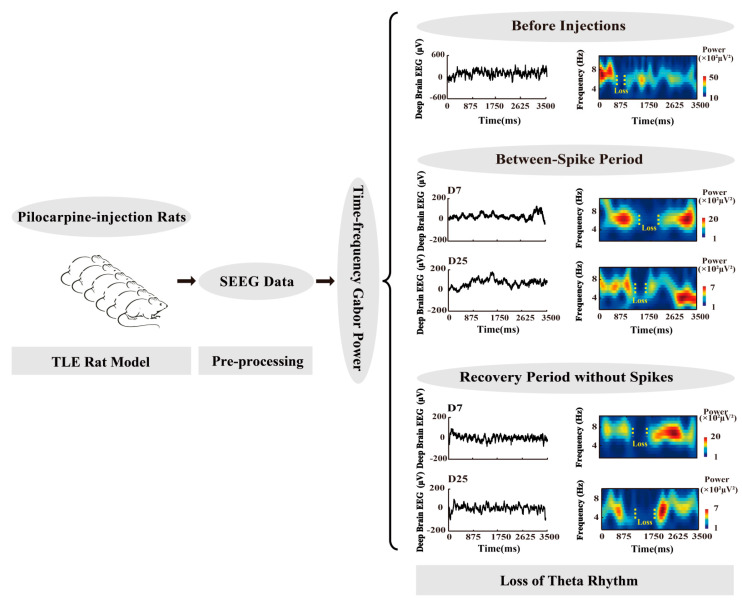
Methodology flowchart for this study.

In this study, the deep-brain EEG epochs including interictal spikes while the rat is performing exploratory behavior are collected, since it is the only behavior that displays spontaneous hippocampal theta oscillations in the waking state. Although many complex factors result in loss of theta rhythm [1], the temporal evidence of IAs or ILAs to make the loss of theta rhythm during epileptogenesis is lacking at present. Here, the deep-brain EEG recordings recorded from 13 rats’ pilocarpine-induced TLE during exploring are analyzed. Loss of theta rhythm is estimated by time–frequency power during the period between adjacent interictal spikes and the recovery period without spikes, in a comparison study between early stage and later stage in the rat CA1 region, by considering before injections (BI) as a control. This research might provide a temporary piece of evidence for interictal spike discharges to cause loss of theta power during the time course to lead to epilepsy and, therefore, be helpful to further understand the dysfunctions in theta-related memory in epilepsy. The study flowchart is outlined in Figure 1.

## 2. Materials and Methods

### 2.1. Pilocarpine-Injected Rats and Deep-Brain EEG Recordings in the Rat CA1 Region

**Figure 2:**
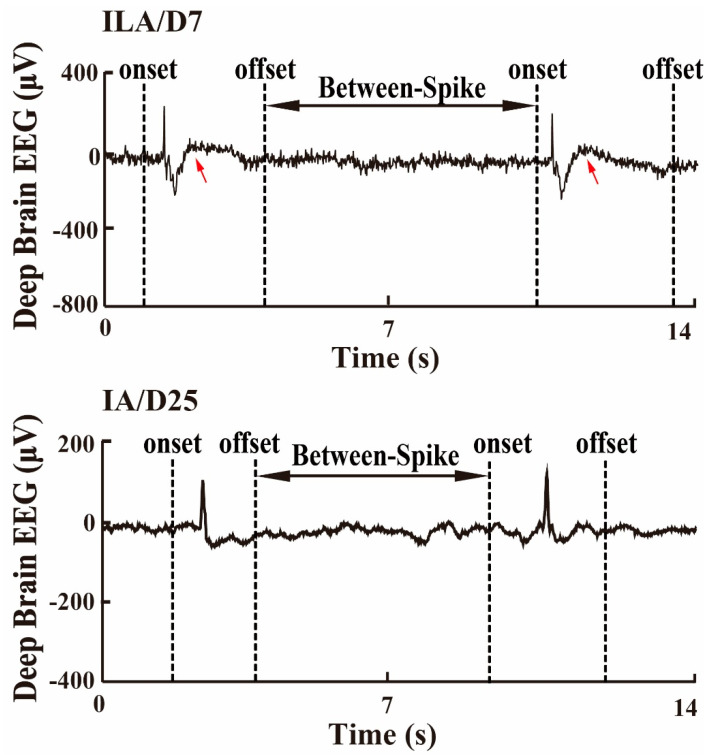
Between-spike period at D7 and D25 on a segment of deep-brain EEG. Red arrow indicates the character of ILA.

Both relevant information about surgical procedures and experimental data of the TLE rat model were provided and reported by French scientists [13]. During operation, electrodes were implanted into a rat CA1 area. Three stainless-steel cortical electrodes were screwed on the skull (two screws in the right and left frontal cortex, and the reference in the cerebellum). When the rats with status epilepticus (SE), a prolonged seizure, were observed for the first time, continuous high-amplitude discharges were also observed on the video-EEG monitoring system (Deltamed^TM^). The deep-brain EEG recordings were recorded on the 7th and 25th days after SE. In the histological study, Day 7 was regarded as latent stage (acute stage, denoted as D7), during which observed spikes were similar to interictal spikes (ILAs) (the up panel in Figure 2). All animals became epileptic by Day 25, which was regarded as the chronic stage (denoted as D25), i.e., when the epileptic discharges were mainly interictal spikes (IAs) (the down panel in Figure 2). After the occurrence of spike bursts, data exhibiting spikes (amplitude at least 10 times larger than that of the background activity) were extracted while the rats were exploring and seizure-free. Following spikes bursts, there were the longitudinal deep-brain EEG recordings recovering free of spikes while the rats were still exploring, whose period was denoted as the recovery period without spikes. Each recording period was taken at least 1 h long. The running speeds of the rats were monitored and recorded, and no obvious anxiety was observed.

### 2.2. Data Pre-Processing

Both data between spikes, as shown in Figure 2, and recovery period without spike were extracted, for the reasons that these data had the prolonged periods, convenient to study loss of theta rhythm near the spike discharges period and the recovery period from the spike discharges. The spike boundary determination was explained in our previous research [14,15]. During each period, epochs were extracted using a 3.5 s time window without overlap. Each 3.5 s epoch was, respectively, defined as a between-spike epoch and a recovery epoch without spikes. Those connotative tiny spikes were excluded from further analyses. The number of spikes, and the longest and shortest period duration, are, respectively, labeled in Table 1.

### 2.3. Loss of Theta Rhythm

Loss of theta rhythm could be estimated using time–frequency Gabor (5-cycle) power and observed directly by the colorful time–frequency map on each 3.5 s epoch to eliminate the edge effect [15]. Then, the map was divided equally into 10 parts (350 ms as a part). For each part, when the background color duration reached 200 ms or more, indicating that theta power (4–8 Hz) suddenly disappeared or became very low (dark blue in the background); the part was denoted as a loss part. Figure 1 illustrates some examples of loss of theta rhythm. The breakage ratio, the ratio of the number of loss parts in a total of parts (%), was employed to elaborate the loss of theta rhythm. In addition, loss of theta rhythm was estimated, respectively, during the between-spike period, recovery period without spikes, and BI. 

## 3. Results

In Table 2, the loss of theta rhythm of all rats is labeled. It was found that loss of theta rhythm during the between-spike period was slightly greater than the recovery period without spikes at both D7 and D25, indicating that loss of theta rhythm was original from the spikes and sustained during the recovery period without spikes. In addition, compared with BI (only 5.81%), loss of theta rhythm was increased by nearly 5 times, compared with that during the recovery period without spike at D7 (27.14%). Moreover, at D25, loss of theta rhythm was sustained but to a less degree, about 6% or about 5% less than D7. The result implied that the role of the interictal spike in loss of theta rhythm was time dependent during epileptogenesis.

Taken together, the emergence of interictal spikes was the direct cause of loss of theta rhythm, and the inhibitory effect of interictal spikes was both persistent and time dependent.

## 4. Discussion

Loss of theta rhythm could cause a decrease in cognitive function. From a macro perspective, theta loss in epilepsy likely involved the interconnected limbic circuits and may disrupt the transfer and integration of information across different brain structures [1]. From a micro perspective, loss of theta rhythm could represent impaired theta networks [12,13], even loss of h channels [16], or some genes such as Na_V1.1_ deficits [17]. Moreover, the early loss of theta rhythm might probably lead to the early spatial memory deficits observed by the same animal models during epileptogenesis [7,13]. 

Changes in synaptic and firing properties of dendrite-inhibiting lacunosum moleculare interneurons could lead to loss of hippocampal theta activity in the mouse model of MTLE [18]. Additionally, from the bodies of evidence surfaced by two animal centers, the dynamics of theta power might be a clinic biomarker for epileptogenesis from the insult to the first spontaneous seizure [19]. At present, there is evidence of interictal spikes that could influence the long-term memory consolidation during sleep in the hippocampus [3]. Finally, the decreased theta power was reported in the spatial memory deficits observed by the same animal models as ours during epileptogenesis [7,13].

When considering the spatial evidence of interictal spike on the loss of theta rhythm provided by our previous research on the TLE patients, that, more loss of theta rhythm was observed in the synchronous IAs genesis areas (e.g., both CA3 and EC) than an independent IA genesis area (e.g., either CA3 or EC) [15]. This research further added temporal proof of the inhibitory impact of interictal spikes on undergoing theta rhythm during the time course to lead to epilepsy. Loss of theta rhythm might be a temporal indicator to qualify the impaired extent caused by ILAs during epileptogenesis. Additionally, loss of theta rhythm was converged to the transient theta power reduction around the spikes in the same rat model during epileptogenesis as in our previous study [14], suggesting a close relationship between loss of theta rhythm during a longitudinal period and transient decreasing in theta power around spikes in epilepsy. Our proof was convergent with the research of [20], indicating that the occurring of ILAs, even soon after SE (i.e., as soon as ILA occurs) could be a direct cause of loss of theta power in TLE.

Future research will explore physical or mathematical modeling to understand the discontinuity of theta oscillations in theory.

## 5. Conclusions and Limitations

We found that loss of theta rhythm became more serious during the period between adjacent interictal spikes than during the recovery period without spike, and during epileptogenesis, more loss occurred at the acute stage than the chronic stage. It was concluded that the emergence of the interictal spike was the direct cause of loss of theta rhythm, and the inhibitory effect of the interictal spike on ongoing theta rhythm during epileptogenesis was not only persistent but time dependent as well, with the time course leading to epilepsy.

There are several limitations to the present study that should be mentioned. To find whether loss of theta rhythm was dependent on the duration of loss part (here, 200 ms), we repeated the analysis by shifting the duration earlier 50 ms (e.g., 150 ms) or later 50 ms (e.g., 250 ms), after which the number of loss parts increased by 5% or decreased by 8%; therefore, our method to estimate the loss of theta rhythm was dependent on the duration of loss part; however, the time-dependent feature of interictal spikes on the loss of theta rhythm was invariant during epileptogenesis.

## Figures and Tables

**Table 1:** Data information during different periods.

Period	Before Injections	Between-Spike Period		Recovery Period without Spikes	
		D7	D25	D7	D25
Number of rats	3 rats	6 rats	5 rats	3 rats	2 rats
Number of spikes	-	224	290	-	-
Longest time (s)	35.38	14.21	12.89	20.37	18.65
Shortest time (s)	7.69	3.7	3.56	5.14	4.96

**Table 2:** Loss of theta rhythm during different periods.

Period	Before Injections	Between-Spike Period		Recovery Period without Spikes	
		D7	D25	D7	D25
Loss number	75	126	906	114	61
Breakage ratio (%)	5.81	30.70	24.30	27.14	22.59

## Data Availability

The data can be provided by the corresponding author upon reasonable request.
